# Neonatal pose estimation in the unaltered clinical environment with fusion of RGB, depth and IR images

**DOI:** 10.1038/s41746-025-01929-z

**Published:** 2025-08-22

**Authors:** Alex Grafton, Joana M. Warnecke, Maxwell Li, Eric He, Lynn Thomson, Kathryn Beardsall, Joan Lasenby

**Affiliations:** 1 Department of Engineering, University of Cambridge, Cambridge, CB2 1PZ UK; 2 Department of Paediatrics, University of Cambridge, Cambridge, CB2 0QQ UK

**Keywords:** Computational biology and bioinformatics, Engineering, Health care, Mathematics and computing, Medical research

## Abstract

Visual monitoring of pre-term infants in intensive care is critical to ensuring proper development and treatment. Camera systems have been explored for this purpose, with human pose estimation having applications in monitoring position, motion, behaviour and vital signs. Validation in the full range of clinical visual scenarios is necessary to prove real-life utility. We conducted a clinical study to collect RGB, depth and infra-red video from 24 participants with no modifications to clinical care. We propose and train image fusion pose estimation algorithms for locating the torso key-points. Our best-performing approach, a late fusion method, achieves an average precision score of 0.811. Chest covering or side lying decrease the object key-point similarity score by 0.15 and 0.1 respectively, while accounting for 50% and 44% of the time. The baby’s positioning and covering supports their development and comfort, and these scenarios should therefore be considered when validating visual monitoring algorithms.

## Introduction

The neonatal period—the first 28 days of life^1^—is among the most critical in human development. Pre-term infants experience increased risk of short- and long-term complications^2–4^, while neonatal mortality accounts for almost half of all under-5 mortality^5^. In the UK, around 100,000 babies—approximately one out of every seven—will spend time in neonatal care every year^6^. In the neonatal intensive care unit (NICU), the highest dependency care, clinical staff are responsible for multiple babies, supported by patient monitors with continuous vital sign monitoring. However, they are unable to provide continuous visual monitoring—each nurse’s attention is divided between patients, while spending time writing notes, preparing feeds and carrying out their other clinical responsibilities. To address this monitoring gap, cameras, coupled with image processing and intelligent decision tools can be employed to identify critical situations^7^.

Continuous pose estimation has a range of applications in supporting neonatal care. Low frame-rate analysis, (e.g. once per second) could monitor the baby’s position and ensure that positioning guidelines^8,9^ are followed. It can also provide a reference point for vital sign monitoring algorithms^10,11^, especially those that rely on a manually selected region of interest^12–14^, or for motion detection algorithms to distinguish localized and gross body movement^15–17^. High frame-rate analysis can provide more detailed information about a baby’s movements, suitable for automatic General Movements Assessment (GMA)^18,19^, cerebral dysfunction, or monitoring sedation^20^.

Existing work in this area is predominantly focussed on controlled environments—well-lit, with babies uncovered and clearly visible, which is ideal for image analysis. In reality, the neonatal incubator can be a visually busy and varied environment, with monitoring equipment, blankets, clothing and nurses’ hands cluttering the scene, making pose estimation difficult. Understanding the performance of computer vision algorithms in this environment is a gap in existing literature. In this paper, we explore several approaches for pose estimation in the clinical environment combining RGB, depth and infra-red imaging. We investigate the covering and position of the babies during the recordings, and evaluate the pose estimation approaches across the different imaging scenarios. Our data includes multiple 24-hour recordings, during which nurses were instructed to continue delivering care as though the camera was not present.

Adult pose estimation is an extensively studied field, summarized by Munea et al. in a review for 2D^21^ and by Wang et al. for 3D pose estimation^22^, and further by Gamra et al. in 2021^23^, and Zheng et al. in 2023^24^. The two primary datasets for adult pose estimation are MPII Human Pose^25^, assessed using the percentage of correct keypoints (PCKh) metric, and MS COCO^26^, assessed using the average precision (AP) metric. The HRNet^27,28^ backbone yields promising results for the MPII dataset^24^, with PCKh@0.5=92.3. In addition, many subsequent state-of-the-art models are based on an HRNet backbone with additional enhancements^29–31^. The vision transformer, specifically ViTPose ViTAE-G,^32^ achieves state-of-the-art performance on the COCO dataset, albeit with vastly more parameters than comparable models. Smaller ViTPose-based models also achieve promising results. Other transformers, such as Swin^33,34^ and HRFormer^35^ are also successful. In our work, we use transfer learning from pose estimation models designed for adults and older infants, which are available in the MMPose model zoo^36^. The MMPose model zoo provides pre-trained models models and training configurations, with readily comparable scores. We use the HRNet model (up to AP=0.767), HRFormer (up to AP=0.774), and ViTPose (sizes S ‘small’ and B ‘base’, AP=0.739 and 0.757 respectively).

Pre-trained models from adults have been frequently used for infant pose estimation and deliver promising results. Huang et al.^37^ achieved an AP of 93.6 using the publicly available infant image dataset MINI-RGBD^38^. This dataset uses synthetic images of infants above term age superimposed on patterned backgrounds. Gross et al.^39^ used 1424 videos from an infant pose dataset and OpenPose^40^ pre-trained on MPII, achieving 99.61% PCKh@0.5, including a mixture of hospital and home recordings. These recordings were also of post-term age infants on plain backgrounds. Moccia et al. use a depth camera in clinical conditions, achieving a median root mean square (RMS) distance of limbs (limbs being a group of three joints) - of 10.2 pixels, across all limbs^41^, and subsequently depth video with temporal processing from 16 preterm infants with a median RMS distance of 9.06 pixels^42^. Further work^43–45^ continues improve neonatal pose estimation, though it is found that generalizability across neonatal datasets is poor. This behaviour is also found by Jahn et al.^46^, who show that retraining on one’s own infant dataset improves performance on that particular dataset, it fails to generalize to other infant datasets where it is outperformed by a generic adult model.

These datasets differ in terms of both the ages of the subjects, ranging from pre-term newborns to old babies, and the recording environment. The BabyPose dataset^47^ contains depth images of babies in incubators, our target demographic, but babies are uncovered and their limbs are clearly visible. The MINI-RGBD dataset^38^ is a synthetic dataset with generated images imposed over real baby poses. Each has a plain background. No existing dataset includes images of babies in the presence of blanket covering or clinical interventions. Understanding the performance of these algorithms in this environment is needed to demonstrate feasibility for continuous clinical use. Datasets used in the literature are summarized in Table 1. We distinguish between an *infant*—a baby who is post-term age, and may be able to move and crawl unaided, and a *neonate*—a new-born baby, who has yet to be discharged from hospital. Only the BabyPose dataset contains neonates in their NICU cots. The work in this paper focusses on *pre-term neonates*.

**Table 1:** Summary of existing infant/neonate image datasets

Dataset	Type	Imaging	Environment	Subjects	Scene
MINI-RGBD^38^	S	RGBD	Flat Surface	Infant	Unclothed, Supine
SyRIP^37^	R+S	RGB	Home	Infant	Varied Poses
BabyPose^47^	R	D	NICU	Neonate	Unclothed, Supine
AGMA^44^	R	Stereo RGB	GMA Clinic	Neonate	Unclothed, Supine
AggPose^43^	R	RGB	GMA Clinic	Infant	Unclothed, Supine
CII-RGBD^45^	R	Stereo RGB	GMA Clinic	Infant	Supine, Varied Clothing
Jahn et al.^46^	R	RGB	GMA Clinic	Infant	Supine, Varied Clothing

*R* Real, *S* Synthetic.

To overcome this challenge, the fusion of different camera types may increase reliability^48^ because each delivers different information which could not be extracted from a single signal^49^. For example, although depth imaging does not see patterns and similar features, it is unaffected by poor lighting conditions. Although existing work has included either RGB or depth images, to our knowledge no work has attempted to combine multiple image types within the same framework.

We recorded 24 babies using a calibrated RGB, depth and IR camera system during 1-hour and 24-hour sessions in the neonatal intensive care unit at Addenbrooke’s Hospital, Cambridge, UK. These recordings were in the normal clinical settings, and the 24-hour recordings included real interventions, light changes, movement and interactions with parents. This paper investigates the performance of signal fusion approaches for combining the three imaging methods in the unaltered clinical environment. In summary, we make the following contributions:

- An assessment of the types of scenes found in the normal NICU clinical environment, and the performance of pose estimation algorithms.
- Proposal and comparison of methods for combining RGB, depth and IR imaging for pose estimation, where earlier stages are separate (image-specific) and later stages are shared.
- Comparison of neonatal and infant datasets, finding that models trained on our dataset show good generalization to other NICU data, but poor generalization to older infant and adult data.

## Results

### Dataset

**Fig. 1:**
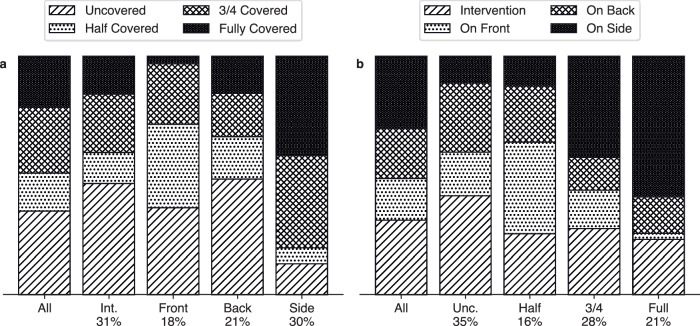
Distribution of the 24-hour data collection, divided into position and covering. **a** Each column represents a given position. **b** Each column represents a level of covering. Int. intervention, Unc. uncovered. Percentages indicate the overall time in that position or covering.

The data collection study consists of our 1-hour recordings, testing the clinical set-up, and 24-hour recordings, which we use to analyse the clinical environment. Each recording contains RGB, depth and infra-red images. The distribution of the baby’s position across the 24-hour recordings is shown in Fig. 1, arranged by position and covering. We find that babies are typically less covered during interventions and that the uncovered/half covered period, when the baby is easily visible, accounts for approximately half of the time. Babies are typically more covered when on their side. The supine, uncovered scenario is around 15% of the dataset (excluding interventions) and around 10% (including interventions). The complete distribution for the 1-hour dataset and the 1-hour plus 24-hour dataset is provided in the Supplementary Information (Supplementary Section 1), Supplementary Figs. 1-3 and Supplementary Tables 1 and 2.

### Model evaluation

In addition to *Single-Image models*, which use only one image type (RGB, depth, or IR), we analyse three fusion methods. Early Image Fusion models (EIF) stack the image along the channel dimension at the input, with up to five color channels (RGB-D-IR), or a combination thereof. Intermediate Image Fusion models (IIF-X) have *X* separate stages that are specific to each image type, after which the feature maps are summed and input to a single set of shared layers. Late Image Fusion models (LIF-X) also have *X* separate stages followed by the remaining shared stages, but the heatmaps from each image are only combined after the key-point head. IIF and LIF fusion methods are tested from *X* = 1 to 4, representing separate/shared division after each of the four blocks of HRNet and HRFormer. All models are trained and tested using five-fold cross-validation. More detailed explanation of the models is provided in Section “Pose Estimation Models”.

**Fig. 2:**
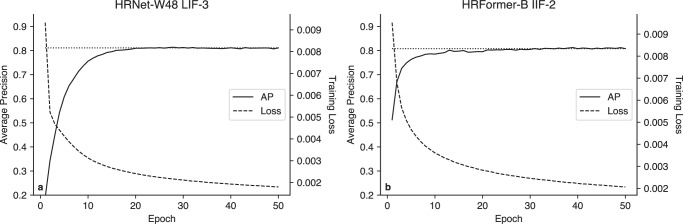
Model training curves showing training loss and test set AP. **a** Training curves for HRNet-W48 LIF-3. **b** Training curves for HRFormer-B IIF-2. The horizontal line shows the final score. In each case, there is minimal difference after 30 epochs.

Table 2 shows the AP scores for the models using the HRNet backbone. Scores are presented as mean ± standard deviation across the five folds. For 10 of the 15 models, models with input size 384 × 384 outperformed those with input size 256 × 256. The HRNet W48 model outperforms the W32 model in 11 of the 15 models. Table 3 shows the scores for the HRFormer and vision transformer backbones. The HRFormer and HRNet models achieve similar scores, though the ViTPose-based models are substantially worse, so several additional experiments are conducted for the depth-only ViTPose-B model to understand if this is due to the experimental configuration. For the depth-only model (AP=0.741), we find that freezing half of the stages reduces performance (AP=0.721) and freezing only the multi-head self-attention module as suggested by the original authors^32^ offers only marginal improvement (AP=0.742). This improved score does not exceed other backbones. Training curves over the 50 epochs are shown in Figure 2 for the HRNet-W48 LIF-3 and HRFormer-B IIF-2 model, with no noticeable improvement on the test sets after 30 epochs.

**Table 2:** AP Scores across model types using the HRNet backbone

Model	W32-256	W32-384	W48-384
RGB	0.753 ± 0.102	0.762 ± 0.095	0.779 ± 0.093
Depth	0.778 ± 0.082	0.769 ± 0.090	0.765 ± 0.091
IR	0.739 ± 0.099	0.747 ± 0.095	0.758 ± 0.096
EIF-RGB-D	0.761 ± 0.086	0.765 ± 0.090	0.768 ± 0.093
EIF-RGB-IR	0.747 ± 0.092	0.765 ± 0.089	0.715 ± 0.121
EIF-D-IR	0.780 ± 0.087	0.760 ± 0.094	0.785 ± 0.083
EIF-RGB-D-IR	0.763 ± 0.088	0.753 ± 0.092	0.773 ± 0.083
IIF-1	0.785 ± 0.083	0.788 ± 0.083	0.793 ± 0.086
IIF-2	**0.800** ± **0.076**	0.784 ± 0.090	0.774 ± 0.094
IIF-3	0.792 ± 0.077	0.790 ± 0.080	0.776 ± 0.094
IIF-4	0.753 ± 0.099	0.761 ± 0.100	0.771 ± 0.097
LIF-1	0.769 ± 0.088	0.775 ± 0.091	0.798 ± 0.078
LIF-2	0.757 ± 0.101	0.775 ± 0.089	0.771 ± 0.095
LIF-3	0.775 ± 0.089	**0.802** ± **0.077**	**0.811** ± **0.069**
LIF-4	0.749 ± 0.099	0.765 ± 0.100	0.772 ± 0.089

Scores in bold are the best scores for each backbone.

*E/I/LIF* Early/Intermediate/Late Image Fusion.

**Table 3:** AP Scores using the HRFormer and ViTPose backbones

Model	HRFormer-S	HRFormer-B	ViTPose-S	ViTPose-B
RGB	0.747 ± 0.112	0.760 ± 0.099	0.714 ± 0.098	0.693 ± 0.129
Depth	0.788 ± 0.097	0.795 ± 0.091	0.657 ± 0.143	0.671 ± 0.139
IR	0.764 ± 0.093	0.776 ± 0.088	0.657 ± 0.131	0.711 ± 0.108
EIF-RGB-D	0.764 ± 0.086	0.783 ± 0.077	0.663 ± 0.076	0.698 ± 0.073
EIF-RGB-IR	0.770 ± 0.089	0.795 ± 0.077	0.645 ± 0.096	0.682 ± 0.077
EIF-D-IR	0.788 ± 0.087	0.795 ± 0.081	0.662 ± 0.14	0.671 ± 0.14
EIF-RGB-D-IR	0.780 ± 0.090	0.777 ± 0.079	0.653 ± 0.082	0.690 ± 0.079
IIF-1	0.785 ± 0.096	0.794 ± 0.094	**0.751** ± **0.105**	0.714 ± 0.100
IIF-2	**0.800** ± **0.096**	**0.809** ± **0.085**	0.715 ± 0.096	**0.753** ± **0.121**
IIF-3	0.785 ± 0.098	0.804 ± 0.088	0.710 ± 0.110	0.746 ± 0.118
IIF-4	0.753 ± 0.103	0.759 ± 0.095	0.721 ± 0.093	0.742 ± 0.091
LIF-1	0.752 ± 0.103	0.752 ± 0.103	0.671 ± 0.096	0.676 ± 0.110
LIF-2	0.772 ± 0.097	0.772 ± 0.097	0.661 ± 0.092	0.674 ± 0.103
LIF-3	0.764 ± 0.105	0.761 ± 0.104	0.625 ± 0.106	0.643 ± 0.106
LIF-4	0.741 ± 0.107	0.744 ± 0.096	0.655 ± 0.093	0.679 ± 0.101

S indicates small configuration, B indicates base configuration. The best performance for each backbone is given in bold.

### Inference time

**Fig. 3:**
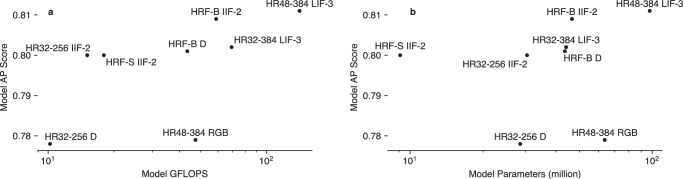
Model performance and computation requirements for selected models. **a** Score against model inference cost. **b** Score against number of parameters. HRF-S/B HRFormer Small/Base, HR32/48 HRNet-W32/48, D Depth.

The inference cost and number of model parameters are given in Fig. 3. Table 4 shows the inference latency in milliseconds for each model across three hardware specifications, reflecting different possible applications: MacBook Air (Apple M2 processor, CPU), for processing on a cot-side iPad or mini PC; a desktop computer with a modest GPU (NVIDIA RTX 3060Ti, CUDA), acting as a local computer away from the cot; a high-performance server (NVIDIA A100 80GB, CUDA) for off-site analysis. Models suitable for real-time (for continuous motion analysis) and 1 frame-per-second inference (for position summaries or vital sign monitoring) are highlighted, thought it should be noted that an off-site server would likely process data from multiple cameras at once time. Results are given for multiple batch sizes where applicable. The HRNet-W48 LIF-3 model achieves the highest performance, at the cost of the highest number of parameters and alongside the HRFormer-B IIF-2 model, the highest inference complexity. The ViTPose models, whose performance is worse in each case than the other models, are not included in these plots. The HRFormer-S and HRNet-W32 IIF-2 models offer high performance at lower complexity.

**Table 4:** Inference latency in milliseconds for selected models in three hardware configurations

		Apple M2	RTX 3060Ti		NVIDIA A100		
Backbone	Model	B=1	B=1	B=10	B=1	B=10	B=100
HRNet-W32-256	Depth	**129**	**11.4**^**a**^	**3.79**^a^	**29.4**^**a**^	**2.94**^**a**^	**0.956**^**a**^
HRNet-W32-256	IIF-2	**316**	**18.2**^**a**^	**14.7**^**a**^	**29.6**^**a**^	**3.96**^**a**^	**3.20**^**a**^
HRNet-W32-384	LIF-3	**208**	**12.5**^**a**^	**5.87**^**a**^	**36.2**	**3.64**^**a**^	**1.52**^**a**^
HRNet-W48-384	RGB	**629**	**31.7**^**a**^	**24.6**^**a**^	**91.8**	**8.87**^**a**^	**6.53**^**a**^
HRNet-W48-384	LIF-3	1070	**51.7**	**44.8**	**89.9**	**11.9**^**a**^	**9.59**^**a**^
HRFormer-S	IIF-2	**432**	**23.3**^**a**^	**16.5**^**a**^	**55.9**	**7.60**^**a**^	**6.49**^**a**^
HRFormer-B	Depth	**784**	**52.4**	**40.0**	**47.0**	**16.6**^**a**^	**15.0**^**a**^
HRFormer-B	IIF-2	1090	**59.3**	**52.0**	**57.0**	**21.2**^**a**^	**19.2**^**a**^

The three hardware configurations represent a cot-side small PC, on-site desktop PC, or off-site server.

Bold indicates suitability for 1fps inference.

*B* batch size.

^a^30fps inference.

### Effect of position and covering

**Fig. 4:**
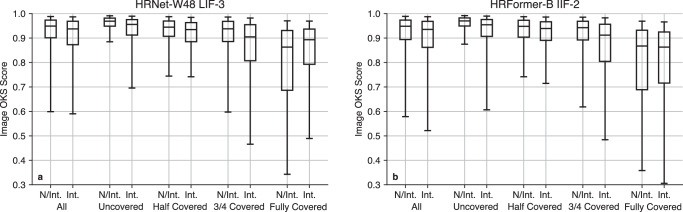
Box-whisker plots of object key-point similarity (OKS) scores, divided by level of covering and intervention. **a** Scores are shown for the HRNet-W48 LIF-3 model. **b** Scores are shown for the HRFormer-B IIF-2 model. I Intervention, N/I Not intervention. Quartiles and 5/95 percentile are shown.

Next, we consider variations in the level of covering and presence of intervention in the image for the best performing models. The OKS scores for a selection of the best performing model backbones and architectures are presented in Table 5 divided by position and covering. For every model, including those not presented in this table, increasing the covering decreases the mean OKS score, most notably when the baby is fully covered. The differences in performance between every pair of coverings or positions for each model are significant at *p* = 1*e* − 5, except for prone/supine. The prone and supine positions are similar in performance and neither position is better overall. However, performance is lower in all models during interventions, and even lower when the baby is on their side. The scores with and without intervention are presented for the HRNet-W48-384 LIF-3 and HRFormer-B IIF-2 models in Fig. 4. Interventions marginally decrease the score on average, and noticeably reduce the fifth percentile score for the HRFormer-B model. The most substantial difference caused by interventions is for babies that are at least 3/4 covered. This may be expected given that interventions effectively increase the level of covering by occluding parts of the baby, and moving from 3/4 covering to full covering (during non-intervention) drastically reduces performance. All differences between covering/intervention are significant at *p* = 1*e* − 5 except uncovered/half-covered with interventions, 3/4-covered/fully covered with interventions, and half-covered with/without interventions. The range of OKS scores is presented, rather than the average precision, as these scores are taken across each fold of cross-validation (and hence with different models). The complete statistical analysis is provided in the Supplementary Information (Supplementary Section 2), comparing covering and interventions in Supplementary Tables 3-5, position in Supplementary Table 6 and covering in Supplementary Table 7.

**Table 5:** Mean OKS score for selected best-performing models

Backbone	Model	Uncovered	Half-Covered	3/4 Covered	Fully Covered
HRNet-W32-256	Depth	0.925	0.898	0.880	0.753
HRNet-W32-256	IIF-2	0.933	0.915	0.892	0.772
HRNet-W32-384	LIF-3	0.938	0.908	0.882	0.805
HRNet-W48-384	RGB	0.923	0.919	0.877	0.695
HRNet-W48-384	LIF-3	0.941	0.916	0.882	0.794
HRFormer-S	IIF-2	0.934	0.910	0.892	0.752
HRFormer-B	IIF-2	0.933	0.918	0.891	0.780

Backbone	Model	Prone	Supine	Side	Intervention
HRNet-W32-256	Depth	0.935	0.934	0.823	0.837
HRNet-W32-256	IIF-2	0.952	0.929	0.836	0.861
HRNet-W32-384	LIF-3	0.932	0.946	0.850	0.877
HRNet-W32-384	RGB	0.933	0.919	0.805	0.846
HRNet-W48-384	LIF-3	0.940	0.942	0.843	0.889
HRFormer-S	IIF-2	0.938	0.930	0.824	0.866
HRFormer-B	IIF-2	0.944	0.940	0.835	0.866

Each row shows the score for each level of covering or position. For every model, the score decreases as the covering increases. There is no general trend between prone and supine, but every model scores lower during interventions than prone/supine and lower still when in the side position. Note that mean OKS scores are typically higher than the AP value.

Table 6 shows the comparative performance of the models with and without pre-training on the COCO adult dataset. The models with pre-training achieve higher scores in each case.

**Table 6:** AP Scores for selected HRNet-W32-256 IIF with and without pre-training

Model Type	With Pre-training	Without Pre-training
IIF-1	0.772	0.647
IIF-2	0.781	0.614
IIF-3	0.772	0.645

Each model is trained using either the weights from the COCO dataset, or randomly initialised.

### Cross-dataset comparison

Next, we compare results across other datasets, summarised in Table 7. To create models suitable for grayscale inputs, the first convolutional layer is summed along the input channel dimension. Models trained on COCO perform well on the SyRIP dataset, but poorly on our dataset, indicating that are images are out-of-domain for the adult model. When evaluating models trained on COCO on depth images, both BabyPose and our dataset perform poorly; this is expected as the model is expecting grayscale images which would contain different features. We use the HRNet-W48-384 model to compare performance on other datasets. We find that our model performs well on BabyPose, outperforming the original work, while performing poorly on SyRIP; this can be expected due to SyRIP’s images being of older babies, and noting the high score for COCO → SyRIP models. We also see that training on our dataset has greatly degraded performance on the COCO dataset (AP=0.698 to 0.351 for RGB and AP=0.682 to 0.081 for depth).

**Table 7:** Cross-domain model performance

Dataset	AP	AP50	AP75	RMSE
COCO (RGB)	0.698	0.832	0.738	–
COCO (RGB) → SyRIP	0.727	0.931	0.810	–
COCO (RGB) → Ours (RGB)	0.223	0.426	0.197	–
COCO (Gray)	0.682	0.800	0.729	–
COCO (Gray) → BabyPose	0.201	0.377	0.202	–
COCO (Gray) → Ours (Depth)	0.118	0.230	0.106	–
Ours (Depth)	0.765	0.944	0.850	7.11
Ours (Depth) → BabyPose	0.824	0.979	0.951	5.68
Ours (Depth) → COCO (Grayscale)	0.081	0.214	0.060	50.84
BabyPose*	–	–	–	9.06
Ours (RGB)	0.779	0.929	0.837	–
Ours (RGB) → SyRIP	0.383	0.639	0.395	–
Ours (RGB) → COCO (RGB)	0.351	0.588	0.360	–
SyRIP*	0.911	0.985	0.985	–

A → B indicates a model is trained on A and tested on B. Models labelled * have scores taken directly from the respective authors’ original work. The RMSE is only included for depth models to allow comparison with BabyPose authors’ results.

**Fig. 5:**
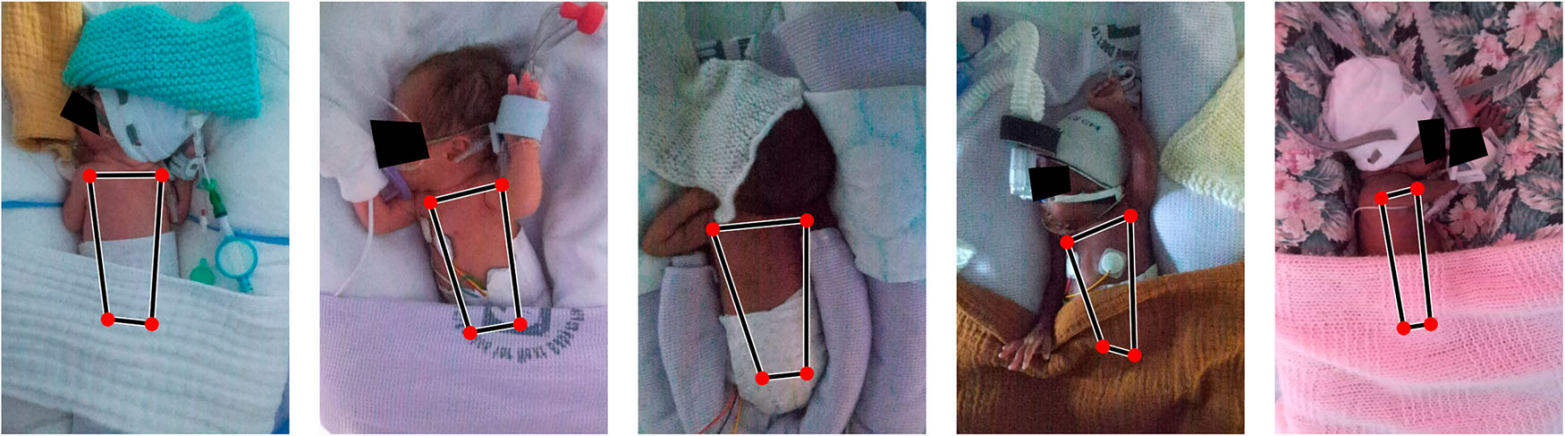
Example images, one from each of the five dataset folds. Each image is shown with the detections overlaid. In the fourth image, the model is inaccurate when locating the hips due to the baby being on its side.

**Fig. 6:**
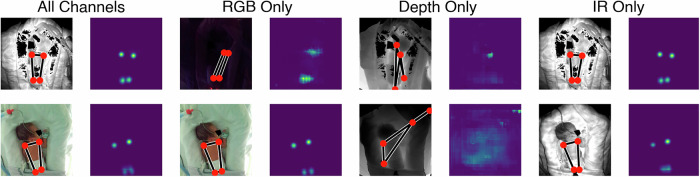
Detections for example images when only one image type is available. In each case, the other images are set to zero. The input image and summed heatmaps are shown. The top example is for a dark image (covered incubator). The bottom row shows a well-lit image.

Finally, we present some examples of images in our dataset, generated using the HRNet-W32-256 IIF-2 model. Figure 5 shows examples of infants in different poses with key-point detections. Figure 6 shows two examples, where only one image type is provided with the others set to zero. In the first example, the image is very dark and the model only works well when the IR image is given. In the second example, the image is well-lit and the model works best when the RGB image is available, although the IR image works well, but providing only the depth image to the fusion model does not allow good detection. This approach is tested across the entire dataset, presented in Table 8. The IIF-2 models lose performance when any images are missing, though the performance loss is lowest for the depth image. For the LIF-3 model, the performance loss is much smaller when images are missing and removing the depth image marginally increases performance. Poor visibility is the most common scenario affecting image quality. Some examples of motion artefacts are provided in Supplementary Fig. 4 and discussed in the Supplementary Information (Supplementary Section 3).

**Table 8:** AP scores for selected fusion models when an image is not provided, or when a single image is provided

		All	Missing Image			Single Image		
Backbone	Model	Images	RGB	Depth	IR	RGB	Depth	IR
HRNet-W32-256	IIF-2	0.800	0.675	0.734	0.716	0.494	0.088	0.164
HRNet-W48-384	LIF-3	0.811	0.741	0.813	0.744	0.749	0.064	0.746
HRFormer-S	IIF-2	0.800	0.662	0.753	0.704	0.500	0.015	0.151
HRFormer-B	IIF-2	0.809	0.608	0.771	0.724	0.496	0.004	0.104

## Discussion

The challenges of the neonatal clinical setting necessitate an alternative approach from typical RGB pose estimation. This paper has proposed early, intermediate and late fusion methods for combining RGB, depth and infra-red images for neonatal pose estimation, based on retraining from models originally trained for pose estimation in adults. Four model structures were explored; the use of a single image type only, or combining images at the input stage (EIF), summing features within the model (IIF) or summing output heatmaps (LIF). The possibility of sharing weights for each image type was also considered.

The dataset contained 14 one-hour and ten 24-hour recordings. The 24-hour recordings were analysed to understand the percentage of time that babies spend in different positions and coverings. It was found that babies spent approximately half of their time in the uncovered (no blanket cover) or half-covered (blanket covering the lower half of the body) state. In the remaining half of the data, the majority of the baby’s chest is covered. A subset of frames were annotated for training, with increased frequency where the depth variation was greater. While this approach somewhat biases the dataset towards selecting images with interventions, the number of frames selected in a fifteen-minute period is fixed; babies’ positions and coverings change much less frequently. Excluding interventions, 31% of images contained babies on their back (supine), 25% on their front (prone) and 44% on their side. These results have implications for future neonatal camera-based approaches: existing work includes babies which are predominantly unclothed and supine, accounting for around 15% of the non-intervention data in our dataset. This would inflate performance scores as the baby is (a) more visible and (b) always known to be in the same position, so the model does not have to identify if the baby is prone or supine.

We only annotate the hips and shoulders as these joints are visible in most frames. Including other key-points would either unbalance the dataset (joints such as ankles and knees are often not visible) or not represent the NICU environment, as certain types of images would become oversampled. When comparing to other work, only these four joints are evaluated. Five-fold cross validation was used for training and testing. This method ensures that models are tested on every image and baby in the dataset^46^. It also ensures that each baby is tested entirely unseen. This should provide greater confidence that the model can perform well on additional data.

We believe that existing methods for scoring detections require some adaptation for neonatal data. The RMS error does not account for the size of the image or subject. The PCKh metric has not been adjusted for the proportionally larger head of neonates, compared to adults. We calculated the median torso-to-head-size ratio for the MPII (adult) dataset (1.28) and for ours (0.83), indicating that the head is larger than the torso for our neonates. The AP metric is chosen as it uses the area of the subject, and contains per-key-point constants to account for the relative difficulty of each joint type. However, the per-key-point constants are based variance of human annotation in images of adults. An appropriate set of constants is needed for neonatal data; for exampe, Jahn et al.^46^ report greater error between human annotators and pose estimators for the hip joints than shoulders. This would require a larger dataset with all key-points annotated, which is a limitation of our work.

Comparison with other work is difficult due to lack of availability of images and models for privacy reasons. In addition, datasets contain images of infants in different environments and at different ages, and no available dataset contains multiple imaging modalities. We are therefore limited to comparing our single-image models with other work. We found that an adult COCO HRNet model with AP=0.682 scores only 0.201 on the BabyPose dataset, and 0.118 on our dataset, while scoring 0.727 on the SyRIP dataset. Conversely, our HRNet models score highly on our dataset (0.779) and the BabyPose dataset (0.824), but lower on the SyRIP (0.351) and COCO (0.383) datasets. The AP scores for our models evaluated on BabyPose (0.824) and ours (0.765) for the depth-only model also suggest that our depth dataset is similar to BabyPose, albeit with more challenging images due to the lower score and inclusion of multiple poses, interventions and covering. In summary, we find generalizabilty between adult/older infant datasets (COCO and SyRIP), and between neonatal datasets (ours and BabyPose), with retraining required between the two types.

Three backbones are tested - HRNet, HRFormer, and the Vision Transformer (labelled ViTPose). For single images, the best performance was achieved for RGB or depth models. A selection of fusion models (EIF-D-IR, IIF-1,2,3 and LIF-3) also performed well, outperforming the single-image models. The best-performing single-image model used the depth image; however, providing only the depth image to the fusion models greatly reduced performance. As the depth and IR images come from the same sensor, it is likely that either only the RGB image is available, or only depth and IR images are available, and in these cases, an RGB-specific or EIF-D-IR model is superior. Despite attempts to modify the training, the vision transformer models exhibited worse performance than the HRFormer and HRNet models.

For the HRFormer-based models and HRNet-W32-256, we find that IIF-X outperforms LIF-X for all X, and that IIF-2 outperforms all EIF models. This is also true for vision transformer models, but the lower overall performance of these models should be addressed before drawing a conclusion. The HRNet W32/48-384 models show different behaviour; LIF-3 outperforms all other models, and also has a marginally lower standard deviation across folds. This difference cannot be attributed purely to the resolution of the image (possibly requiring more modality-specific stages) as the HRFormer models use the same resolution. The LIF-3 model behaves differently to IIF-2 when one or two images are missing; removing the depth image marginally increases the score (0.811 to 0.813) and removing the IR or RGB image does not cause such drastic performance loss. The IIF-2 model’s deterioration can be explained by the latter stages of the model being trained to assume that all images are available, while removing images from the LIF-3 model would simply reduce the heatmap intensity. For example, when the RGB image is dark, the IIF-2 model should rely on the depth and IR images - but when these are removed, performance is poor. Further analysis is provided in the Supplementary Information (Supplementary Section 4) as a test image is made artificially darker (Supplementary Fig. 5). In all cases, only providing the depth image yields extremely poor performance (AP < 0.1), suggesting that fusion models trained on all three modalities learn to rely on the RGB and IR images and not depth.

These models have considerably different inference times - HRNet-W32-256 IIF-2 can be run at 30fps for 8 babies simultaneously on a desktop-class GPU, while HRNet-W48-384 LIF-3 would require a server-class GPU for just three babies. In scenarios where depth or infra-red imaging is unavailable, the LIF-3 model does not show as much performance degradation as the IIF-2 model, and its inference time could be greatly reduced by only evaluating branches for the available images. Further training with missing images may allow the model to become more robust and be used regardless of the imaging configuration. The IIF-2 and LIF-3 models therefore fill different use cases and we would suggest continuing to investigate both.

Using fusion models increases the model complexity, number of parameters and inference time; the later the fusion stage, the greater the effect. The best performing model - HRNet-W48 LIF-3, achieved an AP score of 0.811 but requires over 140 GFLOPS for inference and contains almost 100M parameters. The need for patient privacy and data security may necessitate smaller models for cot-side inference. The HRFormer-S, IIF-2 model requires 18 GFLOPS and only 9.1M parameters, while achieving AP 0.809, which may present a worthwhile trade-off. The best performing single-image model, HRFormer-B with depth images, achieves an AP of 0.801 with 43.3 GFLOPS and 43.6M parameters. We find that using a smaller backbone in a fusion architecture outperforms or matches a larger backbone in a single-image architecture with faster inference and fewer parameters. The HRFormer-S IIF-2 model is suitable for 1 frame-per-second inference on a small, cot-side PC, or real-time inference on a desktop or server PC. It should be noted that including full-body key-point annotations for 24-hour real data is not yet explored and may require the use of larger backbones. This remains a key limitation of this work.

The detection scores are shown to be marginally lower during interventions, and decrease as the baby is more covered. The best performance, with 95% of OKS scores above 0.9, is achieved for uncovered babies without intervention. For fully covered babies, the median OKS score decreases to around 0.86, though the range of scores, as shown in Fig. 4, increases greatly. We find an overall trend of performance decrease as the baby is more covered, regardless of the backbone or fusion architecture.

No conclusion can be drawn on the difference between prone and supine positions; the difference in performance for a given model is not always statistically significant, and the difference is not uniformly positive or negative across models. However, better performance is always seen for prone/supine compared to intervention, and again for intervention compared to the side position. The side position is the most challenging infant position. Despite the side position being more common than the front/back positions, and hence appearing more often in the dataset, it has not caused the models to perform better for babies in this position.

In our 24-hour dataset, a baby spends 44% (excluding interventions) of their time on the side, over 50% at least three-quarters covered and 24% fully covered. We have shown these images to be a more challenging, but real NICU scenario. We also see that for non-intervention images, the range of OKS scores increases as the baby is more covered, indicating that a completely failed detection becomes more likely. In summary, the side position and covered infants are common clinical environments that should be included in test datasets for future neonatal computer vision methods.

The fusion approaches used here simply sum the features from each separate part of the model. Each part’s weight is effectively the amplitude of the feature, so we rely on the separate branches not having large responses to irrelevant features such as a patterned blanket. Other strategies could use a separate part of the model to weight the branches, either on a per-image or per-feature basis. A model like this would require careful training, as plain gradient calculations would give whichever modality is currently favoured by the weighting scheme an effectively higher learning rate. The current fusion schemes are also symmetric. We could use the depth image, currently the best-performing single-image model, which is unaffected by patterns, within an attention mechanism for the RGB or IR images. Image dropout could also be used to make the LIF-3 model into a single model suitable for scenarios where certain image types are unavailable.

We have focussed on a wide range of model architectures and backbones, rather than deep hyperparameter analysis. This would ultimately require a separate validation fold, reducing the available training data. Further work will include more data (from more recordings), annotated with limbs as well as the torso, carefully divided into folds that match the overall distribution of positions and coverings. Once limb key-points have been added, we can also investigate the possibility of reducing the inference time and number of parameters by pruning the model asymmetrically.

This work has focussed exclusively on pose estimation through camera imaging from outside the incubator. The pose estimation accuracy decreases as the infant is more covered and when the infant is on their side. One possible avenue for enhancement involves incorporating additional sensors. Other research has already been conducted to derive movements based on an accelerometer^50^ or electromagnetic tracking systems (EMTS)^51^. It is also possible to use a pressure mat^52,53^, which can provide additional information when the baby is covered, and can confirm or reject spurious pose estimation results.

Improved pose estimation accuracy has significant potential clinical implications for enhancing neonatal care such as providing critical information about an infant when not directly observed by clinical staff. Accurate pose estimation can contribute to the early detection of developmental abnormalities and ongoing monitoring of infant health. These advancements offer valuable insights that can inform clinical decisions and interventions, ultimately improving the standard of care for neonates. This must be considered in the context of the real-world NICU environment; babies are covered for their own comfort, and the supine position is not always ideal for development^54,55^.

## Methods

### Clinical study design

**Fig. 7:**
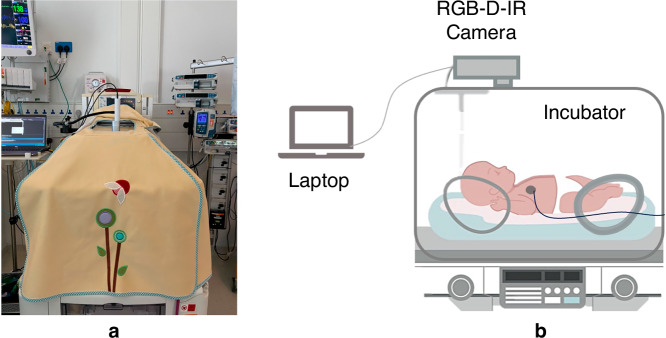
Study recording setup. **a** Experimental setup in the NICU, showing a covered incubator with the camera visible through the hole in the cover. **b** Schematic diagram of the recording setup.

The study was conducted in the Neonatal Intensive Care Unit (NICU) at Addenbrooke’s Hospital, Cambridge, UK, involving 24 preterm neonates in incubators in a single-center study. The study received approval from the North West Preston Research Ethics Committee (reference number 21/NW/0194, IRAS ID 285615) and followed the ethical guidelines of the 1964 Helsinki Declaration. Informed consent was received from each participant’s parent/guardian. Each neonate was recorded for 1 or 24 hours in normal clinical conditions. The incubator environment remained unaltered during the recording. As such, the dataset includes variability in lighting, infant position, and environmental factors. The camera was mounted to the side of the incubator using a flexible arm and placed on top of, and outside, the incubator (Fig. 7). The camera is kept within 1cm of the surface to prevent reflections from the camera’s infra-red emitter, or ceiling lights.

A laptop was used to record and synchronize data from an Azure Kinect camera (Microsoft Corporation, USA) and the neonate’s patient monitor (GE Healthcare Technologies Inc., USA). The Azure Kinect camera captured time-synchronized RGB, depth and infrared (IR) video at 30 frames per second. The RGB resolution was 1280 × 720 pixels, and the depth/IR frames were upsampled to match this resolution from a native 640 × 576 pixels during the RGB/IR alignment. The spatial camera alignment uses the camera’s factory calibration parameters to account for the physical separation between the RGB and IR sensors and their image distortion.

### Dataset

The dataset contains 10 24-hour recordings and 14 1-hour recordings. The 1-hour recordings were during daylight hours and there were minimal interventions and disruptions during the recording. The 24-hour recordings contained the normal range of lighting, interventions and activity, as nurses and parents were instructed to behave as they normally would. The demographic information for the two datasets are given in Table 9, including the mean, standard deviation and range for the gestational age (GA) at birth and recording, weight at birth and recording, and sex/ethnicity. The dataset encompasses a wide range of baby sizes, (485-1950g), GA at birth (23+1 to 31+1 wk+d), and GA at recording (24+6 to 34+6 wk+d).

**Table 9:** Demographic data for neonates included in the study

Description	1-Hour	24-Hour	All
GA (birth) (wk+d)	29+0 ± 19d (24+6,31+1)	25+4 ± 15d (23+1,29+2)	27+4 ± 20d (23+1,31+1)
GA (recording) (wk+d)	31+5 ± 15d (27+2,34+5)	29+6 ± 18d (24+6,34+6)	31+0 ± 18d (24+6,34+6)
Weight (birth) (g)	1068 ± 497 (485,1950)	750 ± 262 (500,1420)	936 ± 438 (485,1950)
Weight (recording) (g)	1289 ± 463 (610,2130)	1088 ± 323 (655,1630)	1205 ± 415 (610,2130)
Sex Ethnicity	12M / 2F 10W / 4NW	7M / 3F 5W / 5NW	19M / 5F 15W / 9NW

Statistics are divided into the 1-hour study, 24-hour study, and combined statistics. Where appropriate, values are given as mean ± standard deviation, (minimum - maximum). Ethnicity is given as white (W) or non-white (NW).

*GA* gestational age.

### Ground truth

Our pose estimation algorithm is designed to locate the torso key-points (shoulders and hips) only. This is because the torso position is visible, or its position can be inferred, in almost all images. Face key-points are often not visible, and limbs are often covered by blankets. By using key-points which can be localized in all images, we have a larger dataset and we can compare the performance in different clinical conditions, such as the level of covering of the infant.

Due to the similarity of consecutive frames, images were annotated manually in 15-30 second intervals using a self-developed annotation tool. In the 24-hour recordings, the mean absolute difference between depth pixels was taken for each fifteen-minute window, and 30 frames were selected so as to be evenly spaced in cumulative depth difference. As such, frames featuring greater activity were prioritised. Annotators selected the position of the shoulders and hips, where they are visible or where they can be inferred. Inferred positions might indicate that although the hip is covered by a blanket, the video may be skipped to a point where the blanket is removed, provided that there is no motion of the baby in that period. Key-points were labelled as visible if they were either clearly visible or under a blanket, occluded if covered by any other object (such as a nurse’s hand), and not visible otherwise. Annotators included clinicians and staff with clinical experience, and multiple annotators reviewed each annotation.

Frames were also annotated with the infant’s position (prone, supine, or on their side), intervention (yes, no), and level of covering (none, half, three-quarter, full). Half covering indicates that the lower body was covered (or a similar amount, for example across the stomach). Three quarter covering indicates that the lower body and a significant part of the torso was covered, but the shoulders are clearly visible. Full covering indicates that the shoulders are only just visible, if at all.

### Pose estimation models

For pose estimation, we use a top-down heatmap approach. As we know each frame contains exactly one baby, we do not need a joint connection method such as associative embedding. The model is built using an HRNet, HRFormer or ViTPose backbone, followed by a key-point head. These models are chosen due their recent state-of-the-art results in adult data, specifically ViTPose and derivatives of HRNet. The models described subsequently are designed to process different image types for the same task. We therefore anticipate that there will be merit to having separate, image type-specific early layers and shared later layers, as we are looking for the same features in each image but the appearance of the features in each image type is different. The HRNet and HRFormer backbones contain input layers followed by four stages. We consider possible separate/shared divisions after each stage; if the division is after the fourth stage, then only the key-point head is shared. The division point is denoted *X*, which can be between 1 and 4. For the Vision Transformer, we determine each stage to be a quarter of the transformer layers, with the patch embedding included in the first stage. We denote the image-specific early stages as *Part 1* of the model and the shared later stages as *Part 2*. We implement four categories of models to process the different image types.

**Fig. 8:**
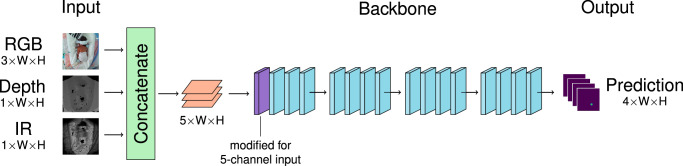
Illustration of the early image fusion EIF-RGB-D-IR model. Images are concatenated along the channel dimension, producing a 5-channel image in the example shown. The very first layer of the backbone is modified to accommodate the different number of channels, and the model proceeds as normal. For other EIF variants (e.g. RGB-D), the number of channels produced would be different.
- *Intermediate Image Fusion Models (IIF-X)*. These models have *X* separate stages, at which point the features are summed before being input to a single set of later stages. If the separate/shared division is after the fourth stage (IIF-4), then the summation is *before* the key-point head. This architecture is illustrated in Fig. 9.

- *Single-Image models* trained and evaluated using a single image type, providing reference performance scores.
- *Early Image Fusion Models (EIF)*. These models concatentate the input images into a single image with up to five color channels (RGB-D-IR), or a combination thereof. This architecture is illustrated in Fig. 8.

**Fig. 9:**
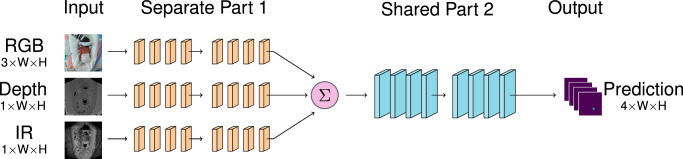
Illustration of intermediate image fusion IIF-X models. Each image is processed through an image type-specific Part 1 with X stages. The resulting feature maps are summed, and processed through Part 2 to give a single prediction.
- *Late Image Fusion Models (LIF-X)*. These models have *X* separate stages, followed by 4 − *X* shared stages to produce a set of heatmaps for each image. The heatmaps are summed (after the key-point head) to produce the overall prediction. These models are the most computationally expensive as they effectively require three models to be evaluated for each frame. This architecture is illustrated in Fig. 10.

**Fig. 10:**
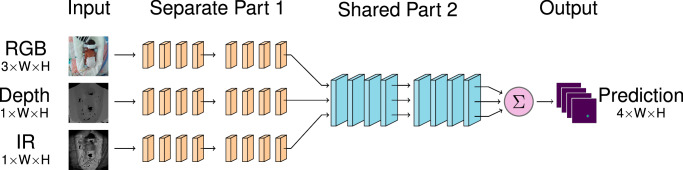
Illustration of late image fusion LIF-X models. Each image is processed through an image-type specific Part 1, followed by a shared Part 2 and the resulting heatmaps are summed.

The models are evaluated for *X* from 1 to 4. We use HRNet backbone configurations W32 and W48 with input image sizes 256 × 256 and 384 × 384. The HRFormer backbone is evaluated at input size 384 × 384 in small and base configurations. The Vision Transformer backbone is evaluated using small and base (S, B) configurations with 256 × 192 image size.

### Evaluation metrics

The COCO average precision (AP)^26^, percentage of correct key-points based on head size (PCKh)^25,56^, and RMS position error are commonly used in the literature for evaluating pose estimation algorithms. We choose to primarily use the AP metric in this work, for the following reasons: we only use four key-points and the AP metric is suitable as it includes a per-key-point constant that accounts for each key-point’s different localisation difficulty; the RMS error is dependent on the image size, which varies across datasets; PCKh is based on the head size of the subject, which does not account for differences in neonatal body proportions, and the difficulty in detecting the different key-points. We also compute the AP metric with/without interventions and different blanket coverage. In the AP metric, each frame *k* is given an object key-point similarity score (*OKS*_*k*_), defined in Equation (1) where *δ*_*ik*_ is the visibility of key-point *i* in frame *k* (1 if visible, 0 otherwise), *d*_*ik*_ is the distance of detected key-point *i* in frame *k* from its ground truth, *κ* is a per-key-point constant reflecting the detection difficulty of each key-point type, and *s*_*k*_ is a scale parameter for frame *k*.

1 $$OK{S}_{k}=\frac{\mathop{\sum }\nolimits_{i = 1}^{4}{\delta }_{ik}\exp (-{d}_{ik}^{2}/(2{\kappa }_{i}^{2}{s}_{k}^{2}))}{\mathop{\sum }\nolimits_{i = 1}^{4}{\delta }_{ik}}$$

Note that detection *k* is equivalent to frame *k* in this work as each frame contains exactly one person. The scale parameter in the COCO dataset is given by the square root of the person’s area. In the absence of the area parameter, the xtcocotools Python^57^ package uses 0.53 × the area of the rectangular bounding box around the person - this factor is approximately the median ratio of person area to bounding box in the dataset. As our dataset does not contain a labelled outline of the baby - this would be impossible due to the blanket - we use the bounding box around the visible key-points to determine the scale parameter, then apply multiplication factors based on the median ratio in the COCO dataset, as follows, depending on key-point visibility (each item’s percentage of our dataset in parenthesis):

1. 3-4 key-points, or 2 opposing corners: use ${s}_{k}^{2}=3.28\times$ the torso bounding box area (95.4%).
2. Only left, or only right key-points: use ${s}_{k}^{2}=2.0\times$ torso height squared (0.9%).
3. Only shoulders: use ${s}_{k}^{2}=6.16\times$ shoulder width squared (3.0%).
4. Only hips: use ${s}_{k}^{2}=13.64\times$ hip width squared (0.7%).
5. If one or zero key-points are visible, the scale cannot be found and the image is not evaluated.

### Training

We employ transfer learning due to the small size of our dataset in comparison with COCO. Pre-trained models are obtained from the MMPose model zoo^36^. Certain weights are adjusted for this application: only indices 5, 6, 11 and 12, corresponding to the shoulders and hips, are used in the key-point head to produce 4 heatmaps instead of 17. For single channel (depth/IR) images, the weights of the initial convolutional layer are summed along the image channel dimension. For EIF models, these weights are stacked along the image channel dimension so that the number of input channels matches the input image. In models with separate stages for each image type, each is initialized with the same initial weights. Before training on our dataset, each pre-trained model was confirmed to produce correct outputs when provided with an image of an adult, with a grayscale version used as a substitute for depth/IR images.

Models are trained for up to 50 epochs using the Adam optimizer method with weight decay^58^ and learning rate 0.0005. The dataset is divided into five folds for cross-validation, and each model is tested on each held-out fold. Each baby’s recording is assigned to a particular fold. Our experiments are implemented in Python (version 3.10) using libraries PyTorch (version 2.3), CUDA (version 12.1), and MMPose (forked from version 1.3.1).

### Cross-dataset evaluation

We compare our results to existing work, namely the SyRIP^37^ and BabyPose^47^ datasets. However, lack of availability of trained models or images, due to participant privacy, makes direct comparison impossible. No other dataset includes all three image types, hence we cannot compare our fusion models. In addition, SyRIP contains images of babies significantly older than those in our dataset, making them not directly comparable. Instead we analyse cross-dataset performance, using models trained on either COCO (adult) or our dataset, and tested on the SyRIP and BabyPose datasets.

Models trained on COCO and their respective evaluation scores are obtained from the MMPose model zoo^36^. These models are converted to grayscale by summing the first convolution kernel along the channel axis, and retaining the axis in the tensor. This is equivalent to converting the image to three-channel grayscale inference. We convert the SyRIP and BabyPose datasets to the COCO format, with only the torso annotated. Images where the torso is not at all visible are excluded. We then evaluate each model on each other dataset. For comparison with the BabyPose dataset, we also evaluate the root-mean-square error (RMSE). We scale the errors to be that of a 128 × 96 image, as used in the original work.

## Supplementary information

Supplementary information

## Data Availability

The datasets generated and/or analysed during the current study are not publicly available due to the sensitive and identifiable nature of the data. The data may be made available for academic purposes under a controlled data sharing agreement.

## Code availability

The underlying code for this study is not publicly available but may be made available to qualified researchers on reasonable request from the corresponding author.
