# Supplementary material for: Neonatal pose estimation in the unaltered clinical environment with fusion of RGB, depth and IR images
Source: NPJ Digit Med. 2025 Aug 22;8:539. doi: 10.1038/s41746-025-01929-z (PMC12373853; doi:10.1038/s41746-025-01929-z)
Supplement: Supplementary file 1 — Supplementary information [file 41746_2025_1929_MOESM1_ESM.pdf]

1 Position and Covering For 1- and 24-hour datasets.

Figures 1-3 show the distribution of scene and position for the entire dataset, 24-hour dataset and 1-hour dataset. The 24-hour dataset distribution figure is identical to that in the main text. The one-hour dataset is clearly different to the 24-hour dataset. The 24-hour dataset (used to analyse the typical NICU environment) is within 2% of the entire dataset (used to train the models).

The raw percentage values used to generate the 24-hour data figure is given in Table 1 (including interventions) and Table 2 (excluding interventions).

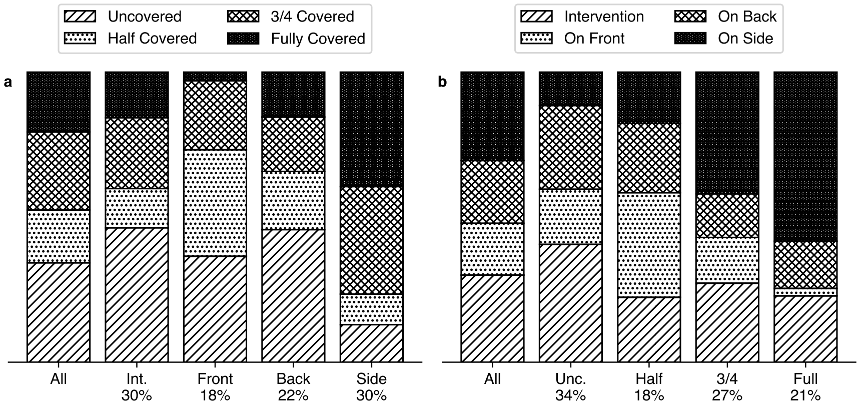

Supplementary Figure 1: **Distribution of the entire data collection, divided into position and covering. a** each column represents a given position. **b** each column represents a level of covering. Int. = intervention, Unc. = uncovered. Percentages indicate the overall time in that position or covering.

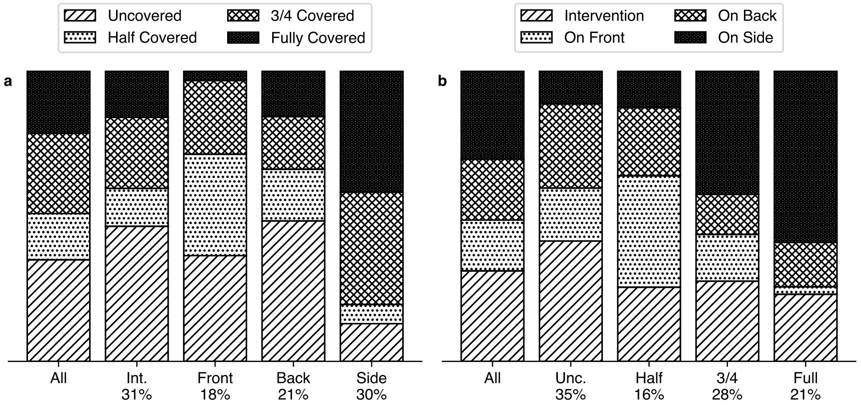

Supplementary Figure 2: **Distribution of the 24-hour dataset, divided into position and covering. a** each column represents a given position. **b** each column represents a level of covering. Int. = intervention, Unc. = uncovered. Percentages indicate the overall time in that position or covering.

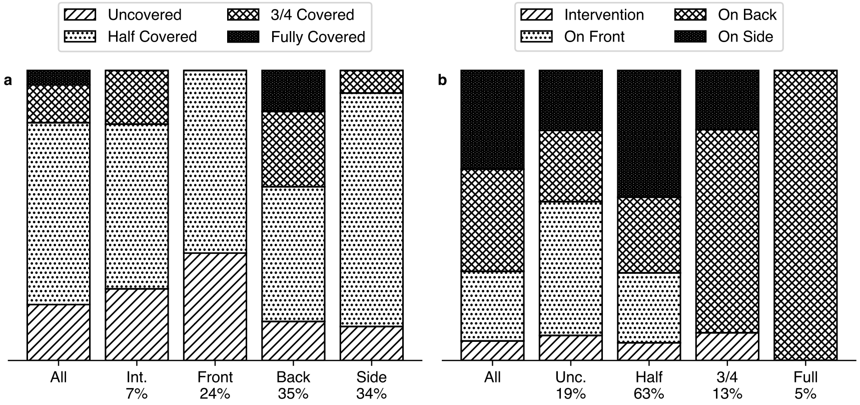

Supplementary Figure 3: **Distribution of the 1-hour dataset, divided into position and covering. a** each column represents a given position. **b** each column represents a level of covering. Int. = intervention, Unc. = uncovered. Percentages indicate the overall time in that position or covering.

Supplementary Table 1: **Percentage of time with each position and covering, with interventions included.**

	Uncovered	Half-Covered	3/4-Covered	Fully Covered	Total
Intervention	14.5	4.1	7.6	4.9	31.2
Front	6.4	6.1	4.5	0.6	17.5
Back	10.2	3.7	3.8	3.3	21.0
Side	3.9	2.0	11.7	12.6	30.3
Total	35.0	16.0	27.6	21.4	

Supplementary Table 2: **Percentage of time with each position and covering, excluding interventions.**

	Uncovered	Half-Covered	3/4-Covered	Fully Covered	Total
Front	9.3	8.9	6.5	0.8	25.5
Back	14.8	5.4	5.5	4.8	30.5
Side	5.7	2.9	17.1	18.3	44.0
Total	29.8	17.2	29.1	23.9	

2 Statistical Significance for Performance across Covering and Position

In the main text, it is shown that the models' performance is lower for babies who are more covered, on their side, or during interventions. The following tables provide evidence of statistical significance of these claims. We use Mann-Whitney U-test to check significance, as the distributions shown in Fig. 3 (main text) are not Normal due to (a) their long tails and (b) their maximum value of 1.0. Some comparisons produce extremely low p-values; any value below 1e-10 is rounded to zero. Tests are performed for covering (HRFormer-B IIF-2, Table 3 and HRNet-W48-384 LIF-3, Table 4), intervention (Table 5), and for a selection of models for position (Table 6) and covering (Table 7).

Supplementary Table 3: **Significance test results for HRFormer-B IIF-2, across levels of covering.** Entries show p-values; values below 1e-10 are rounded to zero.

Not Intervention				Intervention			
	Half	3/4	Full		Half	3/4	Full
Uncovered	0	0	0	Unc.	1.1e-5	0	0
Half-Covered	-	6.5e-10	0	Half	-	0	0
3/4-Covered	-	-	0	3/4	-	-	0

Supplementary Table 4: **Significance test results for HRNet-W48 EIF-3, across levels of covering.** Entries show p-values; values below 1e-10 are rounded to zero.

Not Intervention				Intervention			
	Half	3/4	Full		Half	3/4	Full
Uncovered	0	0	0	Unc.	0	0	0
Half-Covered	-	0	0	Half	-	0	0
3/4-Covered	-	-	0	3/4	-	-	3.3e-4

Supplementary Table 5: **Significance test results comparing intervention vs non-intervention for each level of covering.** Entries show p-values; values below 1e-10 are rounded to zero.

Covering	HRFormer-B IIF-2	HRNet-W48 LIF-3
Uncovered	0	0
Half-Covered	2.7e-4	2.1e-9
3/4-Covered	0	0
Fully Covered	2.2e-6	6.3e-7
All	0	0

Supplementary Table 6: **Significance test results comparing each position across a selection of models.** Entries show p-values; values below 1e-10 are rounded to zero. Int. = intervention.

Model	Front-Back	Front-Side	Front-Int.	Back-Side	Back-Int.	Side-Int.
HRF-B IIF-2	1.2e-2	0	0	0	0	0
HRF-S IIF-2	1.3e-5	0	0	0	0	0
HRNet-W32-256 Depth	4.4e-1	0	0	0	0	2.8e-5
HRNet-W32-256 IIF-2	0	0	0	0	0	0
HRNet-W32-384 LIF-3	0	0	0	0	0	0
HRNet-W48 RGB	3.6e-10	0	0	0	0	0
HRNet-W48 LIF-3	1.7e-1	0	0	0	0	0

Supplementary Table 7: **Significance test results comparing each level of covering across a selection of models.** Entries show p-values; value below 1e-10 are rounded to zero. These results are different to Table 3 and Table 4 are intervention and non-intervention are grouped together.

Model	Unc.-Half	Unc.-3/4	Unc.-Full	Half-3/4	Half-Full	3/4-Full
HRF-B IIF-2	0	0	0	0	0	0
HRF-S IIF-2	0	0	0	0	0	0
HRNet-W32-256 Depth	0	0	0	0	0	0
HRNet-W32-256 IIF-2	0	0	0	0	0	0
HRNet-W32-384 LIF-3	0	0	0	0	0	0
HRNet-W48 RGB	7.1e-2	0	0	0	0	0
HRNet-W48 LIF-3	0	0	0	0	0	0

3 Examples and Effects of Motion Artefacts

Motion artefacts are a common concern for imaging systems. We searched the dataset for three images with visible motion artefacts. No image contained artefacts from gross body movement, likely due to the infant being unable to move the entire body at sufficient speed. Instead, we identified images where limb motion caused the region to appear blurry. We found that these occurred most frequently when the incubator is partially covered.

The images are shown in Figure 4. The blurry motion artefacts are only visible in the RGB images, where the camera's exposure control has increased the exposure time. Although image 1 and 3 appear bright, this is due to longer exposure; this can be seen by the very bright left-hand side of image 1. The depth and IR sensors have a shorter exposure time which is not increased in low-light conditions, as they have an active emitter. They show motion artefacts as regions where the depth cannot resolve appearing as dark patches in the images. The dark patches are smaller than the blurry regions and appear at the baby's hand, where the motion is fastest, rather than along the entire arm. This would indicate that the depth/IR images are more robust to motion than the RGB camera.

Despite the motion artefacts, the shoulder detections are not noticeably affected, though this should be revisited with a model that also detects elbows and wrists. These detections used the HRFormer-B single image models.

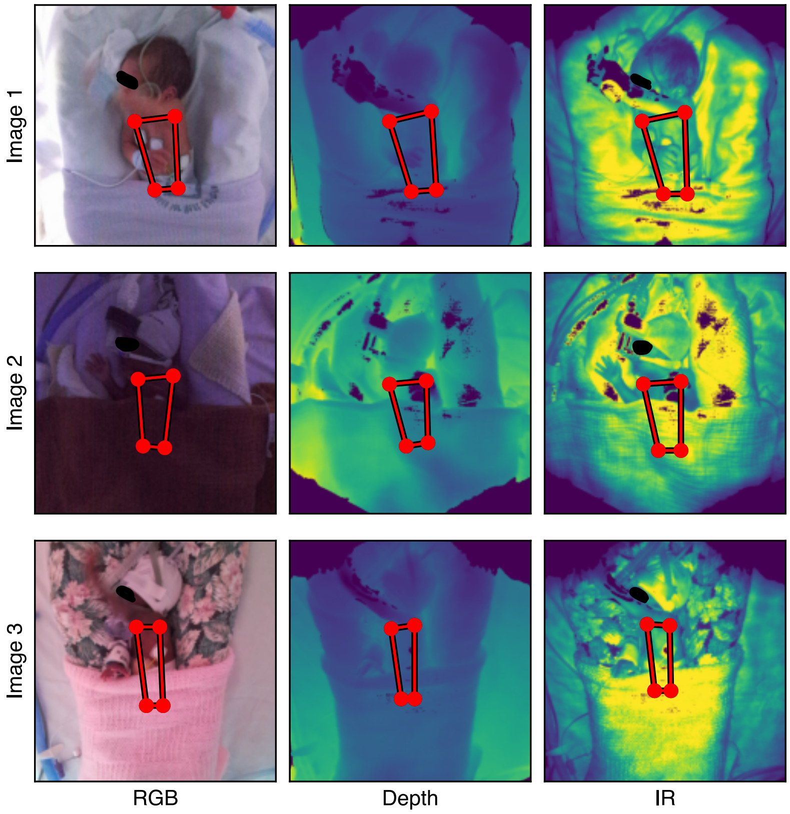

Supplementary Figure 4: **Examples of RGB, depth and IR images and detections. The detections were found using the HRFormer-B single-image models.** Each image has a visible motion artefact during arm movement, appearing blurry in the RGB image and as dark, unresolved patches in the depth/IR images.

4 Examination of IIF-2 Single Image Performance

In the main text, we find that IIF-2 models show poor performance when image types are missing, and offer an explanation. This section includes some additional analysis. We choose a bright image and 'darken' it by adding Gaussian noise (standard deviation linearly spaced from 0 to 150) and then scaling by a factor linearly spaced between 1.0 and 0.1. The noise is added to represent the increased exposure time for dark environments. These calculations are done in floating-point arithmetic, clipped, and converted back to 8-bit pixel values. The model is tested for each image using the HRNet-W32 IIF-2 model (with all images available), the same model with only the RGB available, and the RGB only model.

Figure 5 shows the OKS score and heatmap intensity for the three models. The IIF-2 model is mostly unaffected by the image being darker, as the depth and IR images are available. As the RGB image gets darker, the IIF-2 model would expect to rely on these images - so when they are not available, the OKS score and confidence decrease. The image is shown when the scale is 0.55, around the point where the OKS significantly drops. The RGB model's performance also drops, but only when the image is darker (scale=0.43).

In summary, the IIF-2 model sees a dark RGB image and has learnt not to extract significant features, instead relying on the depth and IR images - so when these images are missing, the detection is poor.

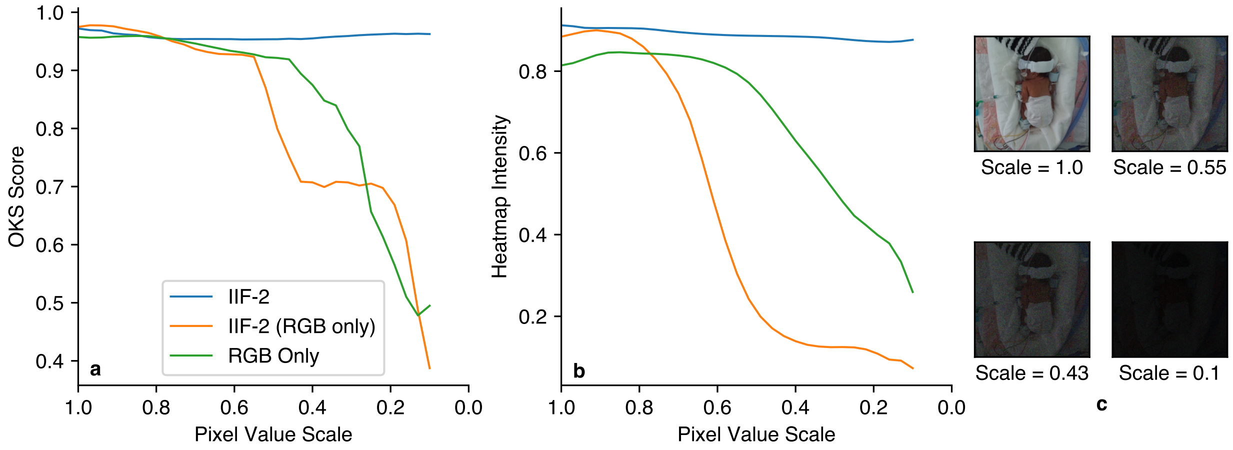

Supplementary Figure 5: **OKS and heatmap intensity as images are made artifically darker. a** The OKS score, representing detection accuracy. **b** The heatmap intensity, indicating the model's confidence. **c** Examples of the images tested, including key image scales where performance decreases. The models used are the HRNet-W32 IIF-2 models (with all images and with only the RGB image) and the HRNet-W32 RGB-only model. The IIF-2 model (without depth/IR) loses detection sooner.
